# Accuracy of Households’ Dwelling Valuations, Housing Demand and Mortgage Decisions: Israeli Case

**DOI:** 10.1007/s11146-021-09823-7

**Published:** 2021-03-06

**Authors:** Alla Koblyakova, Larisa Fleishman, Orly Furman

**Affiliations:** 1 Nottingham Trent University, Nottingham, UK; 2 Israeli Central Bureau of Statistics, 66 Kanfe Nesharim St, P.O.B.34525, 91342 Jerusalem, Israel; 3 Israeli Central Bureau of Statistics, Jerusalem, Israel

**Keywords:** Bias in subjective valuations, House price expectations, Housing demand, Endogeneity, Simultaneous equations

## Abstract

Housing policy, as well as academic research, are increasingly concerned with the role of bias in subjective dwelling valuations as a proximate measure of households’ house price expectations and their relationship with housing demand. This paper contributes to this area of study by exploring the possibility of simultaneous relationships between households’ price expectations and incentive to maximise the size of housing services demanded also accounting for the supply side factors and regional perspective. The empirical estimation takes the form of a system of a two simultaneous equations model applying two stage least squares estimation technique. Cross sectional estimations utilise data extracted from the Israeli Longitudinal Panel Survey (LPS) data. Applying the best available proxy for households’ price expectations, calculated as the ratio between subjective dwelling valuations (LPS) and the estimated market value of the same properties, research has identified the interrelated factors that simultaneously influence householders’ price expectations and housing demand. Results offer conceptual and empirical advantages, highlighting the imperfect nature of the housing market, as reflected by the inseparability of bias in subjective valuations and housing decisions.

## Introduction

Recent housing market developments, and their link with demand for housing in association with accuracy of households’ dwelling valuations (approximate measure for subjective house price expectations), have increased attention directed towards this field of study from both the academic community and policy makers (Brueckner et al. 2012; Campbell 2013; Miles 2012). The existing literature has focused upon the normative implications for housing and mortgage decisions, providing a theoretical basis for discussion, and informing specifications in empirical studies (Duca and Rosenthal 1994; Ioannides and Rosenthal 1994; Leece 2004). Theoretically, households’ excessive valuations and expectations of future price increases result in temporary elevation of house prices (Shiller 2007). This is because overestimation of house value and the expectation of future price increases have a strong impact on demand when households believe any increased value of their home would compensate for investment risks and improve their housing wealth and liquidity position (Case and Shiller 2003; Attanasio et al. 2009). In reality, however, the key question that still needs to be answered is whether the accuracy in subjective dwelling valuations (in addition to changes in fundamentals) has the potential to motivate households’ incentives to maximise housing size and mortgage debt.

This paper addresses this question by exploring whether the bias in subjective valuations and level of housing services demanded are jointly determined, being driven by several interrelated factors. This is with the aim to empirically demonstrate the imperfect nature of the housing market, reflected by the inseparability of households’ expectations and housing decisions. Importantly, given the lack of efficiency in the housing market and the downward momentum in prices associated with overvaluation tendencies, the potential for a significant downward trend in house prices following rapid growth requires attention, while also accounting for regional differences in house prices, the varying degrees of restrictions on new housing supply, land use planning regulations, and local socio-economic conditions (Meen 2008; Shiller 2007; Portnov et al. 2016). This is especially relevant in the Israeli case, where regional deviations across socio-economic clusters, and the various degrees of planning constraints, reflect significant differences in incomes, education, the labour force, housing conditions, house prices and housing supply constraints. The important point to consider is that regional deviations arising from subjective valuations and house price expectations might trigger instability in the housing sector, entrenching regionally differentiated responses to recession. To explore this issue, the current paper accounts for regional effects by including regional dummies and regionally differentiated variables in the empirical estimates.

The empirical modelling framework comprises two consecutive phases. In Phase 1, a separate hedonic model equation was estimated on the base of sale transaction data (2013) provided by the Israeli Tax Authority (ITA), which, in addition to prices of dwellings sold during a given period, provide an assortment of property characteristics. The model’s estimates were used to calculate the market value of dwellings from the Longitudinal Panel Survey (LPS) incorporating data on properties’ physical characteristics. Then, the ratio between subjective dwelling valuations and estimated market value of the same dwelling (bias in subjective valuation), was calculated. In Phase 2, employing the results of the previous phase and applying the household-level data extracted from the second wave of the LPS files (2013), estimation takes the form of a system of two simultaneous equations. The main novelty of the analysis performed herein is the consideration of the imperfect nature of housing markets based on the explicit investigation of simultaneous relationships between bias in subjective dwelling valuations and housing demand, also accounting for supply side factors and the regional perspective.

It is worth noting that the current paper is written at the time when Israel is in national lockdown as a result of the Covid-19 pandemic, which is likely to have significant impact on Israeli housing market, in terms of housing demand, households’ expectations and house price dynamics. As in most other countries, the Israeli government has taken contemporary measures aiming to diminish Covid 19’s impact,^1^ also warning of a significant recession to follow in 2020/21 and beyond, with rising unemployment likely to be pronounced. In the context of the current study, an obvious question to ask is whether understanding of that influencing one of the co-joint determinants of housing demand may simultaneously affect other factors, may contribute in mitigating unexpected consequences associated with the challenges of homeownership decisions.

The paper is structured as follows. The next section analyses the relevant literature; while the third section describes the research data sample. The fourth section details the econometric methodology applied in the paper. The fifth section discusses estimation issues; and the sixth section presents empirical specification. The seventh section provides the descriptive statistics, while the penultimate section presents the findings and interpretation of the results. The final section forms the conclusion.

## Literature Review

### Subjective Dwelling Valuations

The majority of studies on the accuracy of homeowners’ valuations has been based upon comparison between self-reported dwelling valuations and transaction sale prices, i.e., actual prices of the same dwellings if sold during the two years after the survey, or in the year before the survey (Goodman and Ittner 1992; Kiel and Zabel 1999; Benitez-Silva et al. 2009), or with market value estimated using a market-based housing price index (Chan et al. 2016; Davis and Quintin 2017), or a standard hedonic model (Kuzmenko and Timmins 2011). The main criticism of using homeowners’ subjective valuations in surveys is that this approach is prone to problems related to the cognitive complexity of the asset-valuation process (Hurd 1999), and to biases related to survey and item non-response (e.g., Groves et al. 2002). Referring to the specific item of subjective dwelling valuation, Kain and Quigley (1972) found that homeowners who answered an item relating to property value (60% of their sample) were typically those with higher levels of schooling and income, younger age, and less longevity as homeowners. They also found that the response rate among owners of inexpensive properties exceeded that of owners of expensive dwellings. By contrast, Gonzalez-Navarro and Quintana-Domeque (2009) found no relation between item non-response, which occurred in 26% of their sample, and dwelling value or homeowner characteristics. However, some researchers argue that subjective dwelling valuations do not exhibit sample-selection bias, because they reflect price levels more accurately than other data, due to the representative random sample variation of the properties sampled in surveys (Zabel 1999; Steele and Goy 2002; Banzhaf and Farooque 2013).

The main findings of studies on self-reported dwelling valuation have suggested that owner valuations tend to be upward-biased by around 5% on average, although estimates of the bias size fall into a broad range from −2 to 16% (e.g., Kain and Quigley 1972; Robins and West 1977; Ihlanfeldt and Martinez-Vazquez 1986; Goodman and Ittner 1992). It has also been discovered that the bias may vary over time, principally during the periods of significant movement in house prices (Anenberg 2016). For example, Kuzmenko and Timmins (2011) and Chan et al. (2016) provided evidence that households in American cities tend to report higher property values relative to the market prices during downturns, compared to boom market conditions. In support of this, Haurin et al. (2013) reached the same conclusion by comparing list prices with sales prices in the Belfast (U.K.) metropolitan area.

With regard to the reasons for the bias in subjective valuations, various studies have yielded conflicting findings. For example, according to Agarwal (2007), a downward bias is typical for older homeowners and those with higher income, whereas Ihlanfeldt and Martinez-Vazquez (1986), Coate and Vanderhoff (1993), Kiel and Zabel (2008) and Tur-Sinai et al., (2020) found a significant relationship between bias in subjective valuation and homeowner characteristics (age, marital and employment status, income) and property indicators (number of bedrooms, year of construction, parking, and property location). Similarly, Benitez-Silva et al. (2009) found that accuracy in subjective housing valuation correlates with the homeowner’s income and fluctuates with economic conditions in the housing market at the time the dwelling is purchased. Goodman and Ittner (1992), by contrast, found no relation between the characteristics of the property or residential area and bias in subjective dwelling valuation. Gonzalez-Navarro and Quintana-Domeque (2009) found that homogeneous building in a neighbourhood considerably increases the accuracy of a subjective valuation of a property situated in that neighbourhood. There has also been much variation in the awareness of respondents asked to estimate the value of their dwellings relative to the housing market generally, and the price level in the neighbourhood of their dwellings specifically.

Goodman and Ittner (1992), DiPasquale and Somerville (1995), and Gonzalez-Navarro and Quintana-Domeque (2009) found that the closer the purchase of the property was to the survey date, the more accurate the subjective valuation of the dwelling became. By contrast, Zabel (1999) found that longer-term ownership resulted in greater accuracy in dwelling valuation, whereas Agarwal (2007) argued that a shorter term of ownership increases the likelihood of an upward valuation bias. Kuzmenko and Timmins (2011) found that recent homebuyers more precisely assessed the values of local amenities in their subjective dwelling valuations. There is also supporting evidence that households base their housing decisions upon subjective expectations of house price increases, which result from both market-based information and from the delusion that they own a unique property with potential to become extremely valuable in the future (Shiller 2003, 2007).

Within this frame of reference, the accuracy in subjective housing valuations might also be affected by anchoring phenomena. Generally, the anchoring effect refers to the cognitive bias when individuals rely too much on the first piece of information they encounter, or sometimes integrate misleading information into their perception. The power of this phenomenon in affecting human’s perceptions and decision-making is well documented in psychological research (Tversky and Kahneman 1974; Kahneman et al. 1999). Anchoring behaviour is widespread among professional real estate appraisers responsible for assessing both commercial and residential property. For example, Gallimore (1994, 1996) finds significant evidence of heuristic-driven behaviour among residential valuers, arising from a general tendency to formulate an early judgement and subsequently seeking for evidence to justify their assumptions. Moreover, anchoring in residential valuations is believed to be one of the factors influencing not only professional assessors’ valuations, but also subjective homeowners’ valuations. The findings of previous research suggest the anchoring of valuations to past evidence and sales transactions has implications for current valuations (Levy and Frethey-Bentham 2010; McGreal and Taltavull de La Paz 2012; Scott and Lizieri 2012; Tur-Sinai et al. 2020). Therefore, as house purchasing decisions are usually made with little market experience, individuals might “anchor” their valuations to a certain piece of information regarding the housing market, even if it is not up to date or irrelevant.

### House Price Expectations and Housing Demand

The existing literature predominantly views house price expectations as a possible cause of housing demand escalation (Brueckner et al. 2012). Several works suggest that, in addition to demand side factors, the influences of new housing supply need to be considered when referring to households’ adaptive expectations and housing demand. Exploring housing supply effects, Meen (2005) suggests regional and local planning should be more considerate of the housing market signals which can result from households’ adaptive expectations in cases of continuous price increase. Glaeser et al. (2008) raise concerns that any increase in housing demand might be too excessive to be explained only by changes in the fundamentals of the housing market, warning that an underestimation of the role of households’ expectations for future house prices might result in a housing crisis. Important findings reveal that a continuous rise in house prices and growth in housing demand may signal housing market imperfections resulting from insufficient elasticity of the new housing supply and increasingly optimistic household beliefs for future growth in capital gains from housing (Glaeser and Gyourko 2018).

When referring to demand side factors, the dominant argument is that changes in households’ house price expectations, based upon past price appreciation, and to some extent on insufficient elasticity of new housing supply, amplify housing demand and the demand for mortgage debt (Attanasio et al. 2009; Kenny 1999). The resulting increase in demand for housing is then reflected in the market, increasing house price levels (Whitehead and Williams 2011; Tsatsaronis and Zhu 2004**).** One of possible ways to measure the house price expectations is to account for bias in households’ dwelling valuations (Case and Shiller 2003; Niu and Van Soest 2014). The relevant literature emphasizes that house prices are forward looking and dependent on anticipated prices (Poterba 1984). The prospect of being able to sell houses at a higher price in the future, may lead to raising current prices. That is, current house values represent the current discounted value of future price expectations (Towbin and Weber 2015). An overestimation of dwellings’ values by their owners can indicate an exaggerated expectation of house prices, which in turn might cause an actual increase in housing prices, driving both housing and non-housing consumption. “People base life decisions upon vague expectations for the future, and if they have the false impression that they have a unique property that is going to become extremely valuable in the future, then they may consume more … and they may drive up prices today” (Shiller 2007, p. 36). This assumption is supported by findings reported by Case et al. (2012), based on a survey conducted in the number of States in the U.S. during 2006 and 2007. They found that people believe the extremely high prices in the housing market in that period (before the economic crisis in 2008) resulted from homeowners’ overestimations of their property during the preceding couple of years.

When referring to the theory, housing demand is downward sloping but inelastic in price. It may exhibit a rightward shift in responding to an increase in the number of households willing to buy, possibly resulting from a change in expectations, incentives, investment motives, change in income, and demographic factors (Brueckner 1994; Bajari et al. 2013). An important question to answer is through what channels the movement in house prices may influence changes in housing demand levels.

Goodman (2002) and Jones (1994) suggest that the size of housing services demanded is influenced by house price expectations and anticipated levels of housing consumption over the life cycle. The key idea from the life cycle perspective is that income and age should determine the quantity and quality of the housing that households may choose to occupy, given their preferences and ability to pay (Leece 2000; Wilson et al. 2018). That is, later life stage households are less liquidity-constrained and can maximise their utility by buying larger and better-quality houses (Case and Shiller 1990; Englund and Ioannides 1997; Leece 1995; Rosenthal et al. 1991). It has been suggested that the size of accommodation and average house prices could be employed as appropriate proxies for housing demand and user cost of housing services demanded (Brueckner 1994; Tiwari and Hasegawa 2000; Koblyakova et al. 2014).

From a utility-maximisation point of view, households’ housing decisions critically depend on the user cost of owner occupation and differences in the opportunity cost of housing debt (Leece 2000, 2004). It is assumed that rational consumers would try to become cognisant of the level of uncertainty associated with any future house price changes, in addition to changes in incomes, employment status, and housing payments (Goodman 2002). Were such an assessment to be made effectively, then the successful evaluation of the relative risks involved when making a housing decision would produce a high level of utility (Campbell and Cocco 2003).

What also needs to be considered while exploring the drivers of housing demand, is a bias in subjective valuations as an indicator of households’ house price expectations. The ability of the consumer to choose an optimal housing size might be somewhat based on subjective expectations of future house price increases and their degree of financial literacy (Lusardi and Mitchell 2007; Van Rooij et al. 2011). Kaplan et al. (2015) suggest that shifts in beliefs about future house prices could be the dominant force behind the observed swings in housing demand around the Great Recession. The effect of subjectivity on households’ financial decisions is also evident in investment behaviour and mortgage debt decisions (Leece 2004). This is an important factor, as “people are stretching themselves to get on the housing ladder, driven by the twin incentives of hope of capital gains, and the fear of being left behind” (Grannum 2005, p.3). These sentiments, in turn, may create a ‘band-wagon’ effect, shifting demand to the right and amplifying further house price increase. In other words, potential homebuyers may decide to purchase a home that they would normally consider too expensive, because of an expectation that this will be compensated by a price increase. This expectation might also motivate home-buyers to buy sooner rather than later, when they may not be able to afford it (Case and Shiller 2003; Ackert et al. 2011). Overall, self reported dwelling valuations, and expectations of future capital gains are usually based / anchored on former housing price movements, rather than any knowledge of the fundamentals that strongly influence housing demand (Case and Shiller 1988; Campbell and Cocco 2015).

Taken altogether, understanding both fundamental factors and the subjective drivers of housing demand might improve the efficiency of the housing system by informing market participants of the size of their risk exposure and assisting them to detect early signals from the market (Leece 1995, 2006; Koblyakova and White 2017). Of particular importance from a policy perspective is a deeper understanding of the effect of subjective valuation on house prices and the level of housing services demanded. Given that buyers purchase homes infrequently, with a tiny proportion of households active in the market at any one time, even small changes in the aggregate behaviour of a few households could, regionally at least, have substantial influences on house prices and housing demand (Clayton 1996; Alhashimi and Dwyer 2004; Akerlof and Shiller 2010). Moreover, if housing is overpriced for a long period of time, consumers and investors may adjust their expectations upwards, which would influence households’ housing demand (Angello and Schuknecht 2009).

## Research Data Sample

The main research data sample is based on information extracted from the second wave of the Longitudinal Panel Survey (LPS) data files (2013). This survey is the first and only panel survey in Israel to have been conducted annually by Central Bureau of Statistics since 2012, following around 5000 households living in owned dwellings or occupying rented accommodation. The survey focuses on processes affecting households in Israel over time, thereby providing unique information on transitions and changes in various aspects of households’ life cycle stage, economic circumstances and key characteristics of households and individuals in the country.

Since our study focuses on examining the effect of subjective house price expectations that is reflected by bias in self-reported dwellings valuations, the research population comprises only owner-occupier households, accounting for about 61% of the survey population (2818 households) in 2013. The survey data furnishes considerable information about housing, demographic, social, and economic variables including data regarding households’ future income expectations, the ownership of physical assets, and household’s size and composition. The survey question related to dwelling valuations was: “What sum could you obtain if you sold the dwelling today?” The respondent was asked to give a point estimate; i.e., without choosing from a range of values pre-specified on the questionnaire. These records were geo-referenced using a GIS system at the level of census tract. After the geocoding and removal of outlier observations, the research population in the final file totalled 1215 observations (the item response rate for the subjective valuation question equated to over 61%). To account for both macro- and household specific effects, the research data sample also includes information on relevant macroeconomic variables, such as the total population in a locality, the supply of new housing stock, ratio of residential land to number of residents in a locality, and the socioeconomic characteristics of a statistical area. The aggregate level data is assigned to each household using heterogeneity factors, whereas sources of variation originate from the statistical area, locality and geographical district. In addition, an external (ITA) data source (2013) was used to calculate the market value of dwellings from LPS. This source provides information concerning properties’ physical characteristics, in addition to prices of 68,653 dwellings sold during a given period (Israeli Central Bureau of Statistics 2013).

## Methodology

Below we introduce the methodological framework applied in this paper. The estimation procedures comprise two phases. The first phase aims to estimate the market value of dwellings from the survey. This allows us to calculate the bias in subjective valuation as the ratio between subjective dwelling valuations (from the LPS) and the estimated market value of the same properties. As the survey does not provide a direct measure of households’ house price expectations, this ratio became the only available and relevant proxy for the subjective price expectations (Shiller 2003, 2007). The second phase utilizes the results from the preceding phase, comprising a system of two simultaneous equations exploring whether the bias in subjective dwelling valuations and observed ex-post housing demand are simultaneously determined.

### Phase 1: Estimation of the Bias in Subjective Dwelling Valuations

The estimation procedure for examining the accuracy of owner-reported house values as compared to the best available estimate of the house market value (Kuzmenko and Timmins 2011) applies external data source of the ITA transaction data (2013). The estimation procedure begins with a hedonic model based on ITA sale transaction data, which was used to estimate the effect of different property characteristics on its price. Following the theory, hedonic model estimates the effect of the characteristics of the property and its location on property value. These characteristics were determined in accordance with the relevant literature suggesting that the factors related to dwelling price include the structural, physical and location characteristics of the property, such as dwelling floor space, number of floors in the building type of building, year of construction, parking, socio-economic profile of the population in the area and regional data (Arguea and Hsiao 2000; Emrath 2002; Zabel and Kiel 2000). The hedonic model is written in semi log form as follows:

1 $$ {Y}_{ijkl}=1\mathrm{n}\left({P}_{ijkl}\right)={\lambda}_0+{\lambda}_1{Asset}_i+{\lambda}_2{Building}_j+{\lambda}_3C{T}_k+{\lambda}_4 Localit{y}_l+{u}_{ijkl} $$

where *P*_*ijkl*_ denotes the price per square meter of property *i* in building *j* in census tract *k* and locality *l*. *Asseti* denotes the characteristics of dwelling *i* (area, number of rooms), *Buildingj* indicates the characteristics of building *j* (year of construction, building type), *CT*_*k*_ denotes the locational variables of a census tract *k* and residents’ demographic and economic characteristics (socio-economic level of CT, average transactions price), *Locality*_*l*_ indicates the locality characteristics (population, residents’ average annual income, geographical district of locality); and *u*_*ijkl*_ indicates random noise with variance *σ*. A dependent variable ‘logarithm of price per square meter’ and not the actual price per square meter makes it possible to improve the numerical stability of the estimates, reduce their variance, and, as a result, enhance accuracy of LPS dwellings’ value estimation (Epland and Kirkeberg 2012).

After log transformation, the explained variable is approximately normally distributed, justifying the use of the ordinary least squares (OLS) method to estimate Eq. (1). To check for the accuracy and robustness of estimates, the MAPE index has been used (see Appendix 1).

Then, the regression coefficients from a hedonic model were used to estimate the market value of dwellings from the LPS. The ratio between the subjective dwelling valuation and the estimated market value of the same dwelling, the delta variable was defined as follows:

Delta = Subjective Valuation / Estimated Value. This variable is used as a proxy for subjective house price expectations.

### Phase 2: Estimation of Simultaneous Equations Model (SEM)

Applying the results from Phase 1, the modelling approach was focused upon the formulation and simultaneous estimation of two interrelated factors: bias in subjective valuations (proxy for households’ house price expectations) and housing demand. The explicit inclusion of interdependent variables in a system of simultaneous equations aims to detect the imperfect nature of the housing market, as reflected by the inseparability of subjective expectations, and housing and financial decisions.

Thus, Eqs. (2) to (3) represent the formal structure of the model, while Eqs. (4) to (5) represent the empirical specifications of the model, with the variables being used as empirical proxies for the theoretical arguments identified, and in some cases employed for empirical estimations in the previous research (Leece 2004; Brueckner et al. 2012; Goodman 2002; Diaz and Hansz 2007; Case and Shiller 2003).

In these Eqs. (2–3), the modelling explores the simultaneous relationships between the response variables (Yi), and a set of explanatory variables (Xi) that are anticipated to be statistically significant when presented in estimated form. Thus, in Eq. (2), ratio between the subjective dwelling valuation and its estimated market value (Y_1i_) (proxy for households’ house price expectations) is a function of housing demand (Y_2i_) and a set of explanatory variables (X_1i_), with (X_1i_ ≠ X_2i_). In Eq. (3), housing demand (Y_2i_) (proxied by the size of accommodation) is a function of (Y_1i_) and a set of the explanatory variables (X_2i_), with (X_2i_ ≠ X_1i_). In both equations, the first subscript of the parameters indicates the equation number, and the second indicates the variable number. The terms δ and β represent parameter estimates; while (ε_1i_) and (ε_2i_) are random error terms:

2 $$ {Y}_{1i}=\kern0.5em {\delta}_{12}{Y}_{2i}+{\beta}_{11}{X}_{1i}\kern0.5em +\kern0.5em {\varepsilon}_{1i} $$

3 $$ {Y}_{2i}=\kern0.5em {\delta}_{21}{Y}_{1 it}+{\beta}_{22}{X}_{2i}+\kern0.5em {\varepsilon}_{2i} $$

In a two simultaneous equations model, the dependent variables are treated as endogenous, being correlated with the error term in the equation in which they appear as explanatory arguments (Heckman 2008; Heckman and Serletis 2014). This makes the ordinary least squares (OLS) regressor inconsistent, as some effect of the error term is wrongly attributed to the regressor (Amemiya 1974; Wooldridge 2008; Hahn 2001). Resolving an endogeneity problem, the estimation procedure employ an alternative two-stage least squares (2SLS) estimation technique for the bias in subjective valuations equation (Eq. 2) and the housing demand equation (Eq. 3). This methodology is discussed by Angrist and Krueger (2001), Lewbel (2007), Lewbel et al. (2012), Skrondal and Rabe-Hesketh (2014) and Giles and Murtazashvili (2013).

## Estimation Issues

An estimation procedure involves several econometric issues, reflecting specific requirements for the simultaneous equation model (Ebbes et al. 2011; Wooldridge 2010). In particular, a solution to endogenous regressors by applying the 2SLS estimations, require a number of instrumental variables which are used to replace problematic causal variables by calculated values instead of the actual values of the problematic predictors (Amemiya 1974; Angrist and Krueger 2001). A notable issue here is the availability of these instrumental variables, which should be highly correlated with the endogenous regressor and must have zero correlation with the error terms, satisfying relevance and exogeneity conditions (Stock et al. 2002; Wooldridge 2002; Verbeek 2004; Stock and Yogo 2005; Baum et al. 2007). The literature on the instrumental variables approach suggests sources for the selection of instruments derive from theoretical considerations and relevance of instruments’ and exogeneity assumptions (Hahn and Hausman 2002; West et al. 2009). Several tests have been proposed to check if instruments are adequately strong. In this research, the relevance of the instrumental variables was tested using the Rule of Thumb, as detailed by Staiger and Stock (1997), suggesting instruments are adequately strong if the first stage F-statistics exceed 10 for the 2SLS estimates (Verbeek 2000; Hall et al. 1996). In addition, partial R^2^ measures were also used to establish the robustness of the 2SLS estimates (Shea 1993, 1997). Additional checks exploited advantages of STATA software program, applying “robust” STATA commands (Wooldridge 2010; Baum 2007).

The model was identified by applying an exclusion restriction condition; i.e., a necessary and sufficient condition (order condition). In each equation, the number of excluded instrumental variables from one equation was more or equal to the number of endogenous variables included on the right-hand side of the other equation (Greene 2010; Ross 2000; Gujarati and Porter 2008; Wooldridge 2008; Davidson and Mackinnon 1993; Chao et al. 2014). Discussion regarding the identification of the model was presented in the empirical specifications section, while reporting the first stage F-statistics and partial R^2^ results were presented in the discussion of results section.

## Empirical Specification

Econometric specifications provided the basis for the empirical testing of the interdependence between the bias in subjective valuations (proxy for households’ price expectations) and housing demand (proxied as the size of accommodation).^2^ Thus, given the inclusion of the supply side and regional perspective, the empirical specification followed analyses conducted by previous researchers (Brueckner 1994; Brueckner et al. 2012; Leece 2004; Case and Shiller 2003; Campbell and Cocco 2007).

The empirical specifications of bias in subjective valuations is denoted as (*Delta*) and housing demand is denoted as (*Area*); these are given by expressions (4) and (5), respectively.

4 $$ Delta=F\left( Area, Dwelling, Household, Macro, Region\right) $$

5 $$ Area=F\left( Delta, Dwelling, Household, Macro, Region\right) $$

In the case of the two simultaneous-equations model, dependent variables from the left-hand side of one equation concurrently appear as explanatory variables on the right-hand side of the other equations, requiring strong explanatory power (Maddala 1983; Wooldridge 2008). When these variables show statistical significance in both equations, then simultaneity is empirically validated.

For the sake of convenience in presenting the model, we aggregated all explanatory variables in four groups. It should be noted that the variables included in these groups differ across equations. The first group of variables (*Dwelling*) includes property characteristics, while the second set of variables (*Household*) includes personal characteristics of the household, reflecting the life cycle perspective (Brueckner et al. 2012; Leece 2004; Goodman 2002). The third group of variables (*Macro*) includes macroeconomic indicators, such as the city and statistical area characteristics (*census tract, CT*) in which the dwelling is located. These involve socioeconomic profile, locality size (in terms of population), new residential construction, and a proxy for land use regulation and planning constraints. The fourth group of empirical variables (*Region*) relates to regional dummies.

Thus, in Eq. (4), (*Delta*) is a function of the (*Area*), vectors of dwelling (*Dwelling*) and household (*Household*) characteristics, macro variables (*Macro*) and regional (*Region*) dummies. In Eq. (5), (*Area*) is a function of the (*Delta*), vectors of dwelling (*Dwelling*) and household (*Household*) characteristics, macro variables (*Macro*) and regional (*Region*) dummies.

Applying relevance and exogeneity requirements (Hall et al. 1996; Hahn and Hausman 2002), (*Area*) was instrumented by household size (*HH Size*). This variable was excluded from the bias in subjective valuations Eq. (4). The variable (*Awareness*) was used as instrument for (*Delta*), being excluded from the housing demand Eq. (5). The identification strategy employs assumption that family size affects housing demand but does not have a direct impact on the ratio between subjective dwelling valuation and its estimated market value (*Delta*). It is also assumed that household’s awareness (*Awareness*) of housing market conditions and house prices, proxied by actual or expected participation in the housing market due to selling or buying a house, influence households’ housing demand only via that variable’s effect on subjective house price expectations.

For the empirical robustness checks, two additional equations were estimated just for the sample of mortgaged dwellings (approximately 50% of the sample size). In these models, the binary (*Mortgage*) variable was excluded and the ratio between the remaining mortgage debt and the estimated market value of the dwelling (*DVR*) was used instead, aiming to assess the sensitivity of the estimates to the effect of the liquidity constraints and money needed for repaying the mortgage (Leece 2000). The identification of an additional model, comprising two simultaneous equations, was also achieved applying a similar algorithm to that described above.

## Descriptive Statistics

### Key Macroeconomic Statistics

Prior to describing the research data sample, it is instructive to learn about the dynamics pertaining to the key macroeconomic indicators in Israel over a period of five years from 2012 to 2016. Table 1 presented below provides relevant statistics for these macroeconomic factors over time.

**Table 1:** Key macro-economic statistics

Year	2012	2013	2014	2015	2016
GDP (at current prices, NIS^a^ million)	993,441	1,059,101	1,104,746	1,163,769	1,224,168
Percentage of change in GDP compared to the previous year	6.2	6.6	4.3	5.3	5.2
Average monthly wages per employee job (at current prices, NIS)	8971	9212	9373	9576	9799
Percentage of unemployed persons^b^	6.9	6.2	5.9	5.3	4.8
Average dwelling price (at current prices, NIS)	1,142,000	1,232,200	1,308,700	1,392,300	1,463,400
Percentage of change of annual average dwelling price index, compared to previous year	2.6	8.0	6.1	6.4	5.0
Average exchange rate (NIS/US Dollars)	3.86	3.61	3.58	3.89	3.84

^a^*NIS* New Israeli Shekel

^b^Percentage of unemployed persons is calculated of those in labor force

As shown in Table 1, as one moves the time horizon, the economy is seen to improve, indicating a period of economic development characterised by an increase in GDP growth, increase in average wages, and decline in unemployment rates. The outcomes for the housing market reflect a significant increase in prices, peaking in 2013. This necessitates a deeper understanding of what factors drive households’ housing decisions and needs.

### Research Data Statistics

To facilitate the estimation process, the data sample for the research included information on dwellings, the personal and demographic characteristics of the household, new housing supply and planning constraints factors and locality characteristics. The nomenclature and measurements of the variables involved in the estimation process are as presented in Table 2. The descriptive statistics for the variables included in the empirical estimates include the means and standard deviations for the key variables included in the sample.

**Table 2:** Nomenclature of variables and descriptive statistics

Name of variable	Definition of variable	Avg. (S.D.)
Characteristics of household		
HH Size	Number of persons in household	3.64 (1.8)
Ln Income	(Ln) Annual household income (NIS)	11.93 (1.31)
Mortgage	Dummy variable (1/0) for having a mortgage loan in 2013, %	55.5
DVR	Ratio between the outstanding mortgage debt (as of survey day) and the estimated market value of dwelling	0.274 (0.215)
Improvement	Expectations on improving economic situation during 12 months after the survey (Statements: the economic situation of the household will significantly improve or will somewhat improve), %	22.1
Awareness	Dummy variable (1/0) for buying/selling home during 12 months before or after the survey date, %	10.6
Debt	Households’ monthly spending is the same or more than income, %	25.2
Characteristics of head of household		
Male	Male, %	55.7
Age	Age (years)	48.8 (15.3)
Immigrants	Immigrated in or after 1990, %	17.2
Married	Family status: married, %	71.5
Employed	Works for more than 10 months before the survey, %	80.2
Characteristics of dwelling		
Delta	Ratio between subjective dwelling valuation and its estimated market value	1.016 (0.55)
Multistore	Multistore building, %	56
Ln Area	(Ln) Total area of dwelling	4.73 (0.40)
Balcony	Dummy variable (1/0) for a balcony in a dwelling, %	46.1
Parking	Dummy variable (1/0) for a private parking, %	51.2
Storage	Dummy variable (1/0) for a private storage room in the building, %	41.7
Year of construction	Period of building construction, %	
-Before 1947		1.4
−1947-1954		3.2
−1955-1964		5.7
−1965-1974		11.7
−1975-1984		12.4
−1985-1994		17.3
−1995-1999		13.5
−2000-2004		11.5
-after 2005		15.3
Noisy road	Dummy variable (1/0) for noisy road in the vicinity of the building %	35.1
Macro-variables		
Ln ATP	(Ln) Average transactions’ price per square meter in 2013 in census tracts (CTs) in which the dwellings from the LPS are located	9.577 (0.378)
SEC of CT	Socio-economic cluster of CT (see footnote 1) in which the dwellings from the LPS are located	11.57 (3.64)
New construction	Finished new residential dwellings in a locality in 2013 (sq.m.)	146,312 (144,439)
RLR	Ratio of residential land to the number of residents in a locality (sq.m per resident)	66.4 (32.9)
Population	Average population in a locality	166,900 (191,500)

As to the ratio between the subjective valuation and the estimated market value of the same dwelling *(Delta*), the table shows that the average ratio was approximately 1.6%, which falls within the range of findings reported in the relevant literature (“Subjective Dwelling Valuations” section), albeit closer to the lower bound.

The group of homeowner characteristics includes, among others, variable (*Immigrant status),* which refers to immigrants who reached Israel after 1989 (mainly from the former USSR). This population group is distinctive due to its size (approximately one seventh of Israel’s Jewish population in 2009) and the celerity of their arrival (the main immigration wave lasted two years, 1990–1991). The mass arrival of immigrants of common origin, mostly of lower–middle socioeconomic class, strongly affected patterns of home purchase, concentration in specific geographic areas, residential environment, and property values countrywide (Epple and Sieg 1999).

In the group of property characteristics, we defined the (*Balcony)* and (*Parking)* variables as denoting high-importance amenities for a dwelling, given the hot climate in Israel as well as the high density of urban areas, especially in the large central cities. The variable (*Noisy road)* denotes the environmental characteristic perceived by homeowners as a disamenity.

Within the macroeconomic context, average transaction price *(ATP)*, socio-economic cluster *(SEC)* and *(Population)* are used to control for aggregate effects. The effect of an increase in the new housing supply is controlled by incorporating the (*New construction)* factor, proxied by the number of completed new residential dwellings in a locality. To control for planning constraints, the ratio of residential land to number of residents in a locality *(RLR*) serves as a proxy for land use planning regulations and allocation of land resources for residential purpose. Locality effect is controlled for by the inclusion of regional dummies *(REG).*

## Results and Discussion

### Phase 1: Estimation of the Bias in Subjective Dwelling Valuations

In order to calculate the ratio between subjective dwelling valuation and the estimated market value of the same property as that included in the survey, the model for sales transactions was estimated. This calculation employed a conventional hedonic model, which takes the form of a semi-log function, and includes a standard set of principal explanatory arguments; i.e., physical characteristics of the property (such as size of dwelling in terms of room number and area of dwelling, year of construction, type of building), locality characteristics (such as population in a locality, average transaction price per square meter in a census tract, socio-economic cluster) and regional dummies. Note, that the property’s physical characteristics, as described in both the data sources used in the study, ITA and LPS, have a comparable distribution. The same set of property characteristics in ITA and LPS makes it possible to employ the regression coefficients from a hedonic model, based on ITA data to estimate the market value of dwellings from the LPS. To perform the hedonic model estimation, a stepwise selection algorithm was utilised with only variables that were significant to at least the 10% level. The calculated accuracy indices for the estimated model were as follows: MAPE index, 14.54; MedAPE index, 10.24 (Appendix 1). The results of this estimation are presented in Table 3 below.

**Table 3:** Model of sale transaction

Variables	Estimate (S.D)	*p* value
Intercept	2.02 (0.038)	<.0001
No. of rooms	0.10 (0.002)	<.0001
Ln Area	0.49-(0.004)	<.0001
Year of construction:		
1947–1954	0.02- (0.004)	<.0001
1955–1964	0.02 - (0.003)	<.0001
1965–1974	0.01- (0.002)	<.0001
1986–1994	0.04 (0.003)	<.0001
1995–1999	0.08 (0.003)	<.0001
2000–2004	0.12 (0.003)	<.0001
After 2004	0.14 (0.002)	<.0001
Multistore (1/0)	0.17- (0.003)	<.0001
SEC	0.01 (0.000)	<.0001
Ln ATP in CT	0.97 (0.004)	<.0001
Population	*0.00004(0.000006)	<.0001
Haifa District	) 0.010.003)	<.0001
Northern District	0.01 (0.003)	0.027
Central District	0.03 (0.002)	<.0007
Tel Aviv District	0.02 (0.003)	<.0007
R^2^	0.826	
Number of observations	68,653	

As expected, the analysis showed that the dwelling area variable explained most of the variance of the dependent variable (Ln price per square metre), consistent with the findings of previous research (Fleishman and Gubman 2015). Regarding the effect of the year of construction of a residential building on property value, the model showed an upward trend in the regression coefficients over the years. In order to check the effect of the socioeconomic characteristics of population in a CT on property value, an aggregated index was used. It is common practice in the official statistics of several countries (e.g. UK, Australia, New Zealand) to use aggregated indices with the purpose of characterizing and documenting the socioeconomic profile of various geographical units (Burck and Tsibel 2013). The positive effect of socioeconomic profile of CT on property value (Table 3) reflects the well-known correlation between property prices and various effects reflecting the socioeconomic characteristics of the population in a given area (Des Rosiers et al. 2002; Reed 2013; Fleishamn et al. 2015).

Furthermore, there was a strong positive correlation between the dependent variable and the aggregate variable, *(Ln)* mean dwelling prices in CT*,* as a proxy for dwelling prices in the neighbourhood where the specific property is located. The location of the transactions was also controlled for, at both the local level (locality size) and the district level.

After model of sale transactions (1) is estimated for the ITA data and MAPE accuracy test (Appendix 1) has been conducted, the explanatory variable coefficients were used to estimate market value of dwellings from the LPS. The formula used for the calculation of the estimated value is given by the following:

$$ {\hat{P}}_{ikl}=\mathit{\exp}\left({\hat{Y}}_{ijk l}+0.5{\sigma}_{ijk}^2\right) $$

where ${\hat{\sigma}}_{ijk}^2$ denotes the variance of residuals estimated in the regression model.

Then, the ratio between subjective valuation and the estimated market value of the same dwelling, the *(Delta)* variable, was estimated.

### Phase 2: SEM

#### Equation for the Bias in Subjective Dwelling Valuations

Table 4 presents the results from the cross-sectional estimation of bias in subjective dwelling valuations Eq. (4). This was estimated for both the full sample (Model A) and a subsample of mortgaged dwellings (Model B). The estimation procedure employed the two-stage least squares (2SLS) technique.

**Table 4:** Equation for the bias in subjective dwelling valuations. Dependent variable - the ratio between the subjective dwelling valuation and estimated market value of the same dwelling (Ln Delta)

	Model A		Model B	
Explanatory variables	Estimate (S.D)	*P* value	Estimate (S.D)	P value
Intercept	0.36 (0.392)	0.357	0.85 (0.455)	0.064
Ln Area	0.15 (0.050)	0.004	0.13 (0.054)	0.016
Mortgage (dummy; 1 = yes, 0 = no)	−0.04 (0.024)	0.069	–	–
Dwelling characteristics				
Parking	0.06 (0.025)	0.027	–	–
Storage	–	–	0.06 (0.027)	0.025
Noisy road	−0.04 (0.023)	0.094	−0.04 (0.025)	0.086
Homeowners characteristics				
Age Sq	0.00002 (0.00001)	0.057	–	–
Employed	−0.07 (0.030)	0.017	–	–
DVR	–	–	0.24 (0.058)	<.0001
Immigrants	−0.05 (0.030)	0.100	−0.12 (0.030)	<.0001
Improvement	0.03 (0.014)	0.049	–	–
Debt	–	–	−0.05 (0.027)	0.056
Awareness	0.07 (0.036)	0.054	–	–
Macro-variables				
Ln ATP in CT	−0.10 (0.034)	0.004	−0.16 (0.039)	<.0001
Jerusalem District	0.07 (0.043)	0.098	0.10 (0.048)	0.041
Northern District	−0.12 (0.046)	0.021	−0.11 (0.047)	0.021
Number of observations	1215		746	
Mean deviation (pct.)	7		−2	
Adjusted R^2^	0.19 (0.09)		0.22 (0.13)	

Standard errors are in parentheses

Instrumented: Ln (Area)

Instrumental variable: HH Size

First stage F-statistics is 184.9

Shea’s Partial R^2^ is 0.135

When including (*Ln Area*) as an explanatory variable informing the bias in subjective valuations Eq. (4), all estimations show statistically significant values at the 10% significance level for the estimated coefficients.

It was found that (*Delta*) was positively affected by (*Area*) with a coefficient elasticity of 0.146, and negatively influenced by (*Mortgage*) with an elasticity of 0.043. That is, the larger the property a person owned, the more they tended to overestimate the value of their dwelling relative to the property’s market value. The estimation also showed that homeowners who took on a mortgage to purchase their dwelling valued their properties more accurately, compared to those who bought their dwellings on their own. The results also showed that the more amenities properties were equipped with (e.g., storeroom and private parking) the higher the deviations between the subjective dwelling valuations and estimated market values were. By contrast, and as expected, the vicinity of a noisy road to the residential building lowered the subjective property valuation (Nijland and Van Wee 2008). The bias in subjective valuations (*Delta*), was also informed by households’ and homeowners’ characteristics (Brueckner et al. 2012).

In particular, the results showed that unemployed persons typically overvalued their dwellings, while the immigrants’ valuations were closer to the actual market value of their properties (Tur-Sinai et al. 2020).

It was also found that homeowners who expected that their household’s economic situation would improve in the nearest future (12 months after the survey date) tended to overvalue their dwellings (Niu and van Soest 2012).

A possible explanation for this tendency may be found in the domain of economic psychology. Homeownership is one of the most important factors, along with current income, in measuring an individual’s economic well-being. When a person expects an improvement in their household’s general well-being, their subjective standards for the estimation of their proprietary status also increases, thus magnifying the bias in their dwelling valuation (Lewis et al. 1995).

Interestingly, no significant relation was found between household income level and accuracy in dwelling valuation when employment status and well-being expectations were controlled for. The variable (*Awareness*) served as a proxy for a higher household awareness of housing market conditions and house prices compared to homeowners that were not ‘active players’ in the housing market during the periods 12 months before/after the survey date. The estimation results showed that recent homebuyers/sellers or those who planned to buy or sell their property in the nearest future, tended to overestimate the value of their homes, consistent with the findings of previous research (Kiel and Zabel 1999; Agarwal 2007). These results can be better understood in the context of the peak of the house price boom in 2013, empirically confirming that better-informed homeowners tend to overvalue their properties in expectation of further house price increases, thus amplifying housing demand.

Regarding the effect of neighbourhood indicators, the negative estimate of average transaction price in a CT (*ATP*) showed that owners of dwellings in lower price neighbourhoods typically overestimated the value of their dwellings.^3^ The following explanation of this result can be assumed: for homeowners in lower-price localities, the widespread belief that property prices only increase over time may serve as an incentive to overvalue their properties as they generally believe that they will settle their debts by applying the future capital gain that they will obtain by selling their dwelling. In doing so, they tend to accept misleading information that supports their attitudes and beliefs (in an inevitable increase of value of their properties) and disregard contrary information about housing prices in the neighbourhood, developing a bias, akin to a gap between economic and psychological forecasts as documented by Bovi (2009). Another plausible explanation for the overvaluation of property in inexpensive localities is that, as has been shown in research on life- and income-satisfaction, while an increase in an individual’s income and proprietary status improves one’s well-being, having magnified the bias in homeowners’ dwelling valuation, the effect of income in the individual’s reference group (e.g., neighbours) is mostly negative (Easterlin 1995).

When referring to the regional effect, as expected, it was found that in the Jerusalem District (composed mainly of Jerusalem city and several smaller localities) people tended to overestimate the value of their dwellings, while those living in peripheral Northern District typically underestimated the value of their dwellings, ceteris paribus.

To capture the effects of the household’s mortgage repayment relative to the property value, the *DVR* variable was incorporated into empirical estimates (Model B). The inclusion of the *DVR* variable increased the explanatory power of Model (B) compared to Model (A), with R^2^ values of 0.22 and 0.19, respectively.^4^ Overall, it should be noted that there was a certain difference in the mean bias in dwelling valuation in Model (A) compared to Model (B): the contribution of personal indicators to the level of subjective dwelling valuation bias was lower in Model (B). Among the personal indicators that affect dwelling valuation bias, the stability in the variable of immigrant status is notable; its contribution even strengthens in Model (B).

Consequently, one can conclude that, despite the similarity in the models’ results, the factors of having a mortgage and paying off the mortgage do have some effect, both on the size of the subjective valuation bias and on the factors affecting it.

#### Housing Demand Equation

Table 5 presents the results for the housing demand Eq. (5). The estimation procedure employed the two-stage least squares (2SLS) estimation technique. When including the bias in subjective dwelling valuations (*Ln Delta*) and decision to have a mortgage (*Mortgage*) as explanatory variables informing the housing demand Eq. (5), all estimations showed statistically significant values at the 10% confidence level. The results present estimations of the models for both the full sample (Model A) and the supplementary subsample of mortgaged dwellings (Model B).

**Table 5:** Housing demand equation. Dependent variable- housing demand, proxied as area of dwelling (Ln Area)

Explanatory variables	Model A		Model B	
	Estimate (S.D)	P value	Estimate (S.D)	P value
Intercept	4.23 (0.123)	<.0001	4.19 (0.155)	<.0001
Ln Delta	0.34 (0.168)	0.038	0.33 (0.159)	0.037
Mortgage	−0.04 (0.021)	0.087	–	–
Dwelling characteristics				
Multistore	−0.31 (0.024)	<.0001	−0.30 (0.032)	<.0001
Storage	–	–	0.09 (0.031)	0.005
Year of construction				
−1947-1954	−0.21 (0.058)	0.0003	−0.26 (0.075)	0.0005
−1955-1964	−0.13 (0.042)	0.002	–	–
−1999-2004	0.10 (0.035)	0.004	–	–
−2005 or after	0.14 (0.032)	<.0001	0.11 (0.038)	0.004
Homeowners characteristics				
Age	0.003 (0.001)	<.0001	–	–
Income	0.02 (0.008)	0.037	0.03 (0.013)	0.044
HH Size	0.05 (0.007)	<.0001	0.05 (0.009)	<.0001
*DVR*	–	–	−0.11 (0.064)	0.088
Improvement	0.03 (0.012)	0.012	–	–
Macro-variables				
Population	−0.0006 (0.0001)	<.0001	−0.0005 (0.0001)	0.0004
New construction	0.0004 (0.0001)	0.029	0.0007 (0.0003)	0.067
RLR	0.0003 (0.00015)	0.085	–	–
SEC of CT	0.03 (0.004)	<.0001	0.02 (0.004)	<.0001
Tel Aviv District	−0.12 (0.030)	0.0001	−0.08 (0.038)	0.053
Central District	−0.08 (0.031)	0.010	–	–
Haifa District	−0.09 (0.034)	0.006	–	–
Number of observations	1215		746	
R^2^ (Adj.)	0.49 (0.48)		0.46 (0.44)	

Standard errors are in parentheses

Instrumented: Ln (Delta)

Instrumental variable: Awareness

First stage F-statistics is 196.75

Shea’s Partial R^2^ is 0.352

Thus, when (*Ln Delta*) was employed as an explanatory argument, the results showed positive signs suggesting that an increase in the ratio between subjective property valuation and actual market value amplify the demand for housing services. An increase in the likelihood of having a mortgage negatively affects housing size; this may be explained by the liquidity constraints and mortgage costs (Leece 2000; Ambrose and Lacour-Little 2001).

It was also found that the demand for housing in older, multi-storied buildings was lower compared to the most recent attached housing stock. New construction factor (proxy for new housing supply) positively affects the quantity of housing services demanded, possibly reflecting the response to an increase in the supply of housing stock, even given the trend for supplying relatively large apartments (Shiller 2007). Supply side effect is also controlled for with the inclusion of ratio of residential land to the number of residents in a locality (a proxy for land use regulation and planning constraints). Consistent with theoretical considerations, and in-line with previous findings, the coefficient for quantity of land allocated to residential construction per resident has a positive effect on level of housing services demanded (Poterba, 1991; Tiwari 2000; Bajari et al. 2013).

Reflecting life cycle effects and consistent with the theoretical predictions, household income, household size, and homeowners’ age positively affected the size of the desired housing (Jones 1994; Brueckner et al. 2012; Leece 2004). Importantly, expectations of improvement in household’s economic situation tended to amplify housing demand (Shiller 2003). As was expected, the estimation results demonstrated a negative relationship between population size and housing demand, indicating that in larger cities with higher housing density, the size of the housing was smaller. The negative sign of parameter estimates for population may also reflect lower demand when house prices increase.

The parameter estimates for the regional dummies yielded important results. The estimated coefficients for the Tel Aviv District, Central District, and Haifa District showed negative signs, indicating a tendency for a decrease in the size of housing for owner occupation, especially in the Tel Aviv District where the most dense and expensive city Tel Aviv, is located.

The results of the supplementary subsample model (Model B) showed that not just having mortgage debt, but the amount of a household’s mortgage payments relative to the current market value was also negatively correlated to the housing size.

## Conclusion

This paper has contributed to the field of study in three important respects: conceptually, methodologically and empirically. Its conceptual contribution relates to the validation of simultaneous relationships between bias in subjective dwelling valuations and the level of housing services demanded, also considering for the housing supply factors and accounting for the regional perspective. The paper has also identified interrelated factors known to influence bias in householders’ subjective valuations and housing demand. The methodological approach employed a system of two simultaneous equations, applying two- stage least squares estimation technique. Within the SEM framework, this paper identified key factors with the potential to destabilise the housing market, which is a legitimate objective for governments to consider. This is especially relevant in view of the recent house price increase in Israel. The suggestion made here is that government housing policy should account for the subjective drivers of housing and mortgage demand, in addition to drawing on fundamental economic factors.

A deeper understanding of which factors drive excessive increases in house prices might be expected to have important consequences for overall economic performance, since households’ housing decisions might result in investment capital misallocation, by sending the wrong signals to residential investors. The results reported suggest valuable findings regarding regional heterogeneity in the accuracy of subjective dwelling valuations, and thus households’ price expectations within the Israeli housing market. Estimation results illustrate that householders residing in the Jerusalem District are more likely to overestimate their property values and expect an increase in house prices than those living in the peripheral Northern District area. Being an immigrant in the 1990s and being employed, reduces one’s subjective valuations. The increase in the new housing supply and the higher quantity of land allocated to residential construction has the potential to increase demand for the quantity of space. Households’ expectations for a better economic future show the potential to amplify housing demand together with bias in subjective dwelling valuations.

These important findings highlight the imperfect nature of the housing market, as reflected by the inseparability of subjective valuations, and households’ housing decisions. Understanding the issues associated with the efficiency of the housing market in Israel can open an avenue for housing policy measures. This might include an awareness of regional differentiation in the size and risk exposure of households’ housing investment decisions, also assisting in housing policy formulation and design. Possible changes in local property taxes and stimulation of greater elasticity in new housing supply in areas where growing housing demand might result from households’ expectations and demographic shifts, may be also an appropriate option. Assuming that regional and local planning needs to be more responsive to market price signals, policy implications might also include the development of special regionally oriented housing programs, legitimizing land use regulations and planning systems to secure the provision of adequately distributed housing stock. Based upon the conceptual and empirical advantages of this study, policy considerations could also involve the understanding that influencing one of the two simultaneous factors to a certain extent means that other interrelated factors might also be correspondingly affected.

## Appendix 1

To examine estimation quality, the relevant studies use several indices: Mean Absolute Percentage Error (MAPE), Median Absolute Percentage Error (MedAPE), Mean Square Error (MSE), and Forecast Error (FE), to name only the most common. These indices are based on the principle of the measurement of the difference between an assessed value and an actual value. The MAPE index is the most common; the smaller its value is, the more accurate the assessment. The values reported in most studies range from 10% to 35% (Nguyen and Cripps 2001; Lozano-Gracia and Anselin 2012). It is standard practice to calculate these indices on the basis of out-of-sample observations, i.e., those not used in the estimation of the model

To test the accuracy level of the estimated model, the MAPE index is used. It is given by:

6 $$ MAPE=\frac{1}{n}{\sum}_{i=1}^n\frac{\mid {\hat{P}}_i-{P}_i\mid }{P_i}\ast 100 $$

where ${\hat{P}}_i$ denotes estimated value of unit *I* (dwelling) *and* ${\hat{P}}_i$ denotes its actual value, according to relevant data source. In addition, the robust MedAPE index is calculated:

7 $$ MedAPE= Median\left(\frac{\mid {\hat{P}}_i-{P}_i\mid }{P_i}\ast 100\right) $$

To test accuracy, a cross-validation (CV) method with *m* iterations is used. In CV, random sampling is used to divide the original data set into two sub-sets: training sample on which the model is estimated (in the current study, 80% of the original data) and the learning sample on which prediction values and accuracy indices are calculated (the rest 20% of observations). The literature indicates that 10 iterations are sufficient (m = 10) (Efron and Tibshirani 1997). The final estimate is defined as a mean value of those obtained in each individual iteration.

## Footnotes

1 Property tax reduction: https://www.buyitinisrael.com/guide/government-announces-tax-cut-for-real-estate-investors-and-foreign-buyers-of-homes-in-israel/ Deferring loan return: https://www.timesofisrael.com/bank-of-israel-defers-loan-returns-until-end-of-year/ Easing mortgage policy restrictions: https://www.timesofisrael.com/bank-of-israel-announces-major-easing-of-mortgage-policy-restrictions/

2 Within the life cycle theory framework, property size is recommended as an appropriate proxy for housing demand, as the demand for service stream from a housing unit refers to households’ demand for a given quantity of space (Tiwari 2000; Tiwari and Hasegawa 2004; Bajari et al. 2013).

3 The average magnitude of dwellings overestimation (by their owners) in the lowest price neighbourhoods was about 5%, while dwellings’ owners in the highest price neighbourhoods typically underestimated the value of their dwellings by about 2.4%.

4 As for the relatively low R^2^ for bias in subjective dwelling valuations equation (Table 4), we refer to the relevant studies which suggest differentiating between the prediction model and explanatory model. Research addressing individual perceptions, attitudes and behaviour suggests that in the second type of models, relatively low R^2^ coefficient, even less than at the 10% level, is sufficient to provide interesting and important information, and may be of great value when explaining even a small part of the differences in the phenomenon. It is also suggested that the relatively low R^2^ is typical for models like ours, in which the dependent variable is a difference or ratio, rather than the level of a phenomenon (Chin 1998; Neter et al. 1996).
